# Une déshydratation révélant un déficit en 3ßéta Hydroxystéroïde Déshydrogénase: à propos d'un cas

**DOI:** 10.11604/pamj.2015.20.141.5842

**Published:** 2015-02-17

**Authors:** Hanane Latrech, Ahmed Gaouzi

**Affiliations:** 1 Département d ‘Endocrinologie, Faculté de Médecine, Université Mohamed Premier, Oujda, Maroc; 2 Departement d'Endocrinologie Pédiatrique, Faculté de Médecine, Université Mohammed V Souissi, Hopital d'Enfants, Rabat, Maroc

**Keywords:** 3ß HSD, perte de sel, Anomalie du développement sexuel, 3ß HSD, salt loss, sexual development disorders

## Abstract

Le déficit en 3ßéta Hydroxystéroïde Déshydrogénase (3β HSD) est un désordre autosomique récessif rare touchant les voies de synthèse de tous les stéroïdes actifs dans les surrénales et les gonades. Cliniquement, il inclut, à des degrés variables, un syndrome de perte de sel et une hypomasculination des garçons. Ces dernières années, plusieurs avancées en matière de dosages hormonaux et de génétique ont été réalisées ce qui a permis de comprendre les bases moléculaires et le phénotype hormonal de ces patients. Nous exposons à travers un cas clinique les difficultés diagnostiques de ce déficit ainsi que la prise en charge thérapeutique.

## Introduction

Le déficit en 3ßéta Hydroxystéroïde Déshydrogénase (3β HSD) est une forme rare d'hyperplasie congénitale des surrénales (HCS) classée au 3^ème^ rang, par ordre de fréquence, après le déficit en 21 hydroxylase et le 11 β hydroxylase. Cette entité est caractérisée par la présence, à des degrés variables, d'un syndrome de perte de sel, d'une virilisation incomplète des garçons et, chez les filles, d'une virilisation à minima ou des organes génitaux externes normaux [1]. La symptomatologie clinique peut être parfois difficilement reconnaissable découverte à la suite d'un syndrome de perte de sel pouvant mettre en jeu le pronostic vital. Les dosages hormonaux actuellement disponibles peuvent confirmer le diagnostic. Nous rapportons le cas d'un nouveau né âgé de 1 mois qui s'est présenté dans un tableau de perte de sel associé à un micropénis et un hypospadias illustrant les difficultés diagnostiques et la conduite thérapeutique de ce type de bloc enzymatique.

## Patient et observation

**Figure 1:**
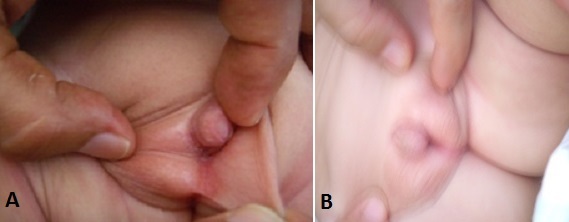
A) organes génitaux externes: hypospadias; B) organes génitaux externes: micropénis

Il s'agit d'un nouveau né de 1 mois issu de parents consanguin de 1^er^ degré consultant pour des vomissements. A l'interrogatoire, on a retrouvé la notion de décès de deux frères à l’âge de un mois dans un tableau de déshydratation aigue. L'examen clinique trouve des signes de déshydratation à type de yeux creux, des fontanelles antérieures déprimées, une bouche sèche et une hypotonie axiale. L'examen des organes génitaux externes révèle un bourgeon génital mesurant 1.5 cm/ 1 cm avec un hypospadias postérieur et des gonades en place (Figure 1). Un ionogramme sanguin réalisé en urgence retrouve une hyponatrémie à 120 mmol/l, une hyperkaliémie à 7 mmol/l, une glycémie à 0.74g/l, une hypochlorémie à 75 mmol/l et un taux de bicarbonates à 14 mmol/l. L’échographie abdominale a révélée une hyperplasie bilatérale des surrénales et organes génitaux externe de type masculins sans présence de dérivés müllériens. Le nourrisson a donc bénéficié en urgence, après réalisation de prélèvements sanguins pour des dosages hormonaux, d'une réhydratation par du sérum salé et d'un traitement par hémisuccinate d'hydrocortisone (6mg/kg/6h). L’évolution était favorable marquée par une disparition des signes de déshydratation et d'une correction des troubles ioniques. Le caryotype sanguin a montré une formule chromosomique 46 XY. Le bilan hormonal a retrouvé ultérieurement un taux de cortisolémie de 8h normal à 19,9 µg/dl (VN: 4.2- 38.4), un taux d'ACTH élevé à 140 000 ng/ml (VN: 10.00- 70.00), un taux de 17 hydroxyprogéstérone supérieur à 20 ng/ ml, un taux de SDHEA élevé à 347 µg/dl, une testostéronémie à 1.09 ng/ ml et un taux de Delta 4 androsténédione à 124,1 nmol/l et un taux d'activité rénine élevé à 3,64 pmol/l (0.2- 2.8). Une génitographie réalisée a objectivé un urètre court de type masculin sans visualisation de vestiges müllériens. Ce tableau clinico- biologique confirme le diagnostic d'un bloc enzymatique en 3β hydroxystéroïde déshydrogénase. On a associé donc à son traitement, en plus de l'hydrocortisone à la dose de 20 mg/m2/j, de la fludrocortisone (50 µg/j). Une cure chirurgicale de son hypospadias a été réalisée après le traitement du micropénis par du Testoviron (25 mg tous les 15 jours).

## Discussion

**Figure 2:**
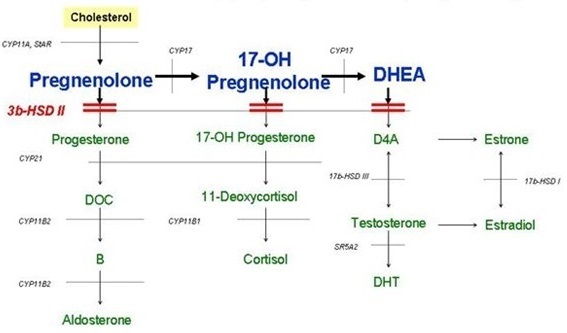
Stéroïdogénèse

Le déficit en 3 ßétaHydroxystéroïde Déshydrogénase (3βHSD) est une forme rare responsable d'environ 1 à 10% des HCS [2]. Ce désordre, autosomique récessif, a été décrit pour la 1^ère^ fois dans sa forme classique en 1962 par Bongiovanni [3]. De 1988 à 1992, date de découverte du gène responsable du déficit en 3βHSD, les études de biologie moléculaires ont permis d’élucider la structure, les fonctions et la répartition tissulaire des enzymes responsables de cette activité 3βHSD. Chez l'homme, il existe deux isoenzymes dimériques de la 3βHSD, le type I et le type II, qui ont 93,5% d'homologie des séquences des protéines [1]. Le type I (3βHSDI) est exprimé dans le placenta et les tissus périphériques. Le type II (3βHSDII) est exprimé, à son tour, dans la surrénale et dans l'ovaire et les testicules. La 3βHSD II est responsable de l'oxydation et l'isomérisation des ▵5 stéroïdes (prégnénolone, 17 OH prégnénolone et la déhydroépiandrostènedione (DHEA)) en delta4 stéroïdes (respectivement, la progestérone, 17OH progestérone et ▵4) [1, 2, 4]. Son déficit complet entraine donc un défaut touchant les trois voies de la stéroïdogénèse surrénalienne à savoir la synthèse du cortisol, l'aldostérone et les androgènes surrénaliens (Figure 2). Ce déficit peut se révéler précocement dans les premiers mois de la vie, par un syndrome de perte de sel avec, chez le garçon et à des degrés variables, une virilisation incomplète des organes génitaux externes (OGE) à type d'hypospadias périnéal ou périnéoscrotal rarement isolé [5] mais souvent associé à un micropénis, un scrotum bifide avec des gonades palpables. Les organes génitaux internes sont normaux [1, 2, 4]. Notre patient a présenté un tableau fait d'un syndrome de perte de sel associé à un hypospadias et à un micropénis. Chez la fille, il n'y a pas d'anomalies des OGE. Cependant, certains cas de virilisation à minima, à type d'hypertrophie clitoridienne, ont été décrits secondaires à la conversion périphérique des précurseurs en androgènes actifs par la 3βHSDI [1, 4, 6]. Dans sa forme non classique, rarement confirmée par biologie moléculaire, le déficit en 3βHSD peut se présenter dans l'enfance par des signes d'hyperandrogénie avec une pubarche précoce associée à une accélération de la croissance staturale puis, en périodes pubertaire et postpubertaire, par un hirsutisme et des troubles de règle [1, 2]. Le diagnostic biologique est fait devant l'accumulation de tous les précurseurs de la voie ▵5 avec une augmentation du rapport ▵5 / ▵4. Une augmentation du taux du 17 OH progestérone, assez bien connue actuellement dans le déficit en 11 βhydroxylase mais aussi en 3βHSD, pourrait être également retrouvée et expliquée par l'existence d'une activité intacte de l'isoenzyme de type I (3βHSDI) capable de métaboliser les taux élevés des stéroïdes ▵5 en stéroïdes ▵4 [1, 4, 7].

Dans les formes avec perte de sel, le taux de rénine est souvent élevé. Chez notre patient, l'association d'un syndrome de perte de sel, qui est une extrême urgence thérapeutique, d'une histoire familiale de décès de deux frères en périodes néonatales et des signes d'hypogonadismes (un micropénis et un hypospadias), nous a permis d’évoquer le diagnostic d'un déficit en 3βHSD qui a pu être confirmé par la suite par la biologie. L'augmentation du taux de la 17OH progestérone, souvent retrouvée dans cette situation, pourrait être à l'origine, chez la fille, d'un retard diagnostique, déjà rapporté dans la littérature, chez des patientes repérées en dépistage néonatal systématique du 21 hydroxylase [4] ou devant l'association de signe de premature pubarche et d'une élévation du taux du 17 OH progestérone [1]. Toutes ces données soulignent l'importance de l’étude moléculaire comme un complément dans la démarche étiologique de ces déficits enzymatiques. Les deux isoenzymes de la 3βHSD sont codées par deux gènes, 3HSD B1 et B2, localisés sur le chromosome 1 en p13.1 [1, 2, 8]. A ce jour, trente neuf mutations du gène 3HSD B2 ont été décrites chez des patients ayant un déficit en 3βHSD II [4, 9] cliniquement symptomatique (perte de sel, virilisation, troubles de cycles et infertilité) [10]. L’étude moléculaire de formes moins sévère de déficit en 3βHSD, définies par un excès des stéroïdes ▵5, n'a pas identifiée de mutations aussi bien au niveau du gène de la 3βHSD [4]. La prise en charge thérapeutique médical de ces patients est principalement substitutive à base d'hydrocortisone associé au fludrocortisone en cas de syndrome de perte de sel, pareil aux autres formes non hypertensives d'HCS [1, 2]. L'adaptation du traitement se fait sur des éléments clinico- biologiques (croissance pondérale et staturale, l’état d'hydratation, le dosage de l'ACTH, les stéroïdes ▵5 et SDHEA [11]. Et en raison de la localisation gonadique de la 3-b-HSDII et donc son rôle dans la synthèse de l’œstradiol, de la progestérone et de la testostérone, la puberté et la fonction gonadique de ces patients peuvent être altérées. Il n'existe que peu de cas décrits dans la littérature. Chez la fille, l’évolution pubertaire est très variable allant d'une absence de développement des seins [12], d'une aménorrhée primaire et une oligoaménorrhée [13, 14] à un développement pubertaire rapporté chez trois patientes [1, 12, 15, 16], aboutissant dans un cas à des menstruations régulières [16]. La puberté et la fonction gonadique des garçons n'ont été décrites à notre connaissance que chez deux patients atteints de déficit sévère en 3b-HSD, l'un dont la fonction de reproduction est normale [8] et l'autre dont la puberté s'est déroulée normalement, mais qui est azoospermique [16]. Le traitement chirurgical de l'hypospadias chez le garçon, fait en général dans la première année de vie, nécessite l'instauration auparavant d'un traitement médical du micropénis afin d’éviter l’échec de la chirurgie. Plusieurs interventions, selon la sévérité de l'hypospadias, sont aussi parfois nécessaires avec un risque de complications postopératoires à type de fistule ou de sténose urétrale.

## Conclusion

Le déficit en 3β HSD est une cause rare, pas toujours confirmée, d'HCS et qui affecte la biosynthèse de tous les stéroïdes actifs dans les surrénales et les gonades pouvant ainsi mettre en jeu le pronostic vital par le risque de survenue d'un syndrome de perte de sel. L'association de ce syndrome de perte de sel à des signes d'hypogonadisme doit faire évoquer assez rapidement le diagnostic chez le garçon et qui sera confirmé assez rapidement par des dosages hormonaux.
